# Production of β-1,3-glucanase and chitosanase from clostridial strains isolated from the soil subjected to biological disinfestation

**DOI:** 10.1186/s13568-019-0842-1

**Published:** 2019-07-23

**Authors:** Atsuko Ueki, Toshiaki Takehara, Gen Ishioka, Nobuo Kaku, Katsuji Ueki

**Affiliations:** 1 Faculty of Agriculture, Yamagata University, 1-23, Wakaba-machi, Tsuruoka, Yamagata 997-8555 Japan; 2 NARO Western Region Agricultural Research Center, Hiroshima, 721-8514 Japan

**Keywords:** Anaerobic bacteria, β-1,3-Glucanase, Biocontrol of soil-borne pathogen, Chitosanase, *Clostridium beijerinckii, Fusarium oxysporum* f. sp. *spinaciae*, Anaerobic soil disinfestation

## Abstract

Biological soil disinfestation (BSD) or anaerobic (reductive) soil disinfestation (ASD/RSD) is a bioremediation method used to eliminate soil-borne plant pathogens by exploiting the activities of anaerobic bacteria in soil. In this study, two obligate anaerobic bacterial strains isolated from BSD-treated soil and identified as *Clostridium beijerinckii* were examined for their abilities to suppress the spinach wilt disease pathogen (*Fusarium oxysporum* f. sp. *spinaciae*) as a representative soil-borne fungal plant pathogen. Both strains degraded β-1,3-glucan and chitosan, two major polysaccharide components of ascomycetes fungal cell wall, supplemented in the medium. β-1,3-Glucanase was detected in the supernatants of cultures supplemented with different types of glucan. Similarly, chitosanase was detected in cultures supplemented with chitosan. Both the enzyme activities were also detected in cultures containing glucose as a substrate. Live cells of *F. oxysporum* f. sp. *spinaciae* that were co-incubated with each anaerobic strain under anaerobic conditions using glucose as a substrate died during incubation. Freeze-dried dead fungal biomass of the pathogen, when added to the culture, supported good growth of both anaerobes and production of both enzymes. Severe and nearly complete degradation of both live and dead fungal cells during incubation with anaerobic bacteria was observed by fluorescence microscopy. When individual anaerobic bacterial strain was co-incubated with live pathogenic fungal cells in wheat bran, a popular material for BSD-treatment, both the strains grew well and killed the fungal pathogen promptly by producing both enzymes. These results indicate that both the bacterial strains attack the fungal cells by releasing extracellular fungal cell wall-degrading enzymes, thereby eliminating the pathogen.

## Introduction

Biological soil disinfestation (BSD) or anaerobic (reductive) soil disinfestation (ASD or RSD) is a method employed to suppress or eliminate soil-borne plant pathogens before starting the cultivation of crops, without using agricultural chemicals (Blok et al. 2000; Goud et al. 2004; Momma 2008; Momma et al. 2013; Strauss and Kluepfel 2015). BSD in a strict sense is a bioremediation technique that uses biological materials and activities of indigenous bacteria inhabiting the soil (Ueki et al. 2018). In BSD treatments, various organic materials (mainly from plant biomass) are incorporated into soil, which is then irrigated with water, followed by covering it tightly with plastic sheets to prevent entry of oxygen for ~ 3 weeks. During the treatment, anoxic conditions in the soil are maintained with rather higher temperature (around 30 °C) under the sunlight. Effectiveness of BSD treatments to kill or inactivate many kinds of soil-borne plant pathogens has been demonstrated (Browne et al. 2018; Butler et al. 2014; Huang et al. 2016; Mazzola et al. 2018; Meng et al. 2018; Messiha et al. 2007; Muramoto et al. 2014; Serrano-Pérez et al. 2017; Shennan et al. 2018; Shrestha et al. 2018). Mowlick et al. (2012, 2013a, b, c, 2014) reported that obligate anaerobic bacteria, mainly various linages from the class *Clostridia*, proliferate in the soil under such treatments. It is expected to clarify the role of anaerobic bacteria thriving in the soil towards suppression of soil-borne plant pathogens. We isolated many anaerobic bacterial strains from soil samples subjected to BSD-treatments (Mowlick et al. 2012, 2013a; Ueki et al. 2017). In the previous study, we reported the ability of representative isolates (H110 and TB8), identified as *Clostridium beijerinckii*, to degrade components related to ascomycetes fungal cell wall (β-1,3-glucan and chitosan) and to kill the fungal pathogen (*Fusarium oxysporum* f. sp. *spinaciae*) under anaerobic conditions (Ueki et al. 2017, 2018). It was strongly suggested that these strains produced extracellular enzymes capable of degrading these components of the fungal cell wall, leading to the destruction of cells. In this study, we investigated the enzymatic activities of the two anaerobic bacterial strains to degrade polysaccharides of ascomycetes fungal cell wall. β-1,3-Glucanase and chitosanase activities were detected in culture supernatants of both the strains cultivated with β-1,3-glucan or chitosan as growth substrates. Fluorescence microscopy using a fluorescent dye (calcofluor white) showed that the mycelial cells of the *Fusarium* pathogen, regardless of whether living or dead, were completely degraded when co-incubated with either of the anaerobic bacterial strains.

## Materials and methods

### Isolation and cultivation of anaerobic bacteria

The anaerobic bacterial strains, H110 and TB8, were isolated from the wheat bran-treated or *Brassica juncea* plant-treated BSD soil samples of the model experiments (treated at 30 °C for 17–18 days) at the NARO Western Region Agricultural Research Center, Hiroshima and at the Tokushima Agricultural Research Center, respectively, in our previous studies in Japan (Mowlick et al. 2012, 2013a). The bacterial strains were isolated and cultivated as described earlier (Ueki et al. 2017, 2018). Briefly, each BSD-treated soil sample was serially diluted (tenfold) anaerobically using an anaerobic dilution solution (Satoh et al. 2002), and 0.2 ml of each dilution was inoculated into 10 ml of anaerobic roll tube agar (Holdeman et al. 1977). After incubation for 7 days, well-isolated colonies were picked up from the roll tube agar and purity of strains was confirmed through repetition of the roll tube method. Strains H110 and TB8 were used as representative strains based on various physiological properties examined (Ueki et al. 2017, 2018). Both the strains were cultivated under anaerobic conditions at 30 °C, unless stated otherwise, using a peptone-yeast extract (PY) medium as a basal medium with oxygen-free mixed gas (95% N_2_ and 5% CO_2_) in the headspace. Each test tube containing the medium was closed tightly with an inner butyl rubber stopper equipped with an outer screw cap. The PY medium contained (per liter) 10 g trypticase (BD BBL, Sparks, USA), 5 g yeast extract, 0.2 g Na_2_CO_3_, 0.3 g l-cysteine·HCl·H_2_O, and 1 mg sodium resazurin, as well as salt solutions (Satoh et al. 2002). The PY medium supplemented with (per liter) 0.25 g of each glucose, cellobiose, maltose, and soluble starch, as well as 15 g agar, designated as the PY4S medium, was used for isolation and maintenance of strains as agar slant cultures. Before autoclaving, pH of all the media was adjusted to 7.2–7.4 with NaOH solution. PY broth medium supplemented with glucose at 10 g/l was designated as the PYG broth and was used for cultivation of the isolates as inoculums for various cultural conditions. Inoculation of pre-cultivated cells to the media was carried out under the stream of oxygen-free mixed gas (95% N_2_ and 5% CO_2_).

Strains H110 and TB8 were identified as *Clostridium beijerinckii* in the family *Clostridiaceae* in the class *Clostridia* in our laboratory (Ueki et al. 2017) and deposited in NBRC (NITE Biological Resource Center, Kisarazu, Japan) as NBRC 112101 and NBRC 112094, respectively.

### Physiological properties of strains H110 and TB8

Physiological examination of the two strains was performed as reported previously (Ueki et al. 2009, 2016, 2017). All the cultivation experiments were performed at least in duplicate and reproducibility of the results was confirmed. Growth of the bacterial strains in the presence of carbohydrates under the anaerobic condition was examined by using the PY broth as a basal medium supplemented with 0.5% or 1% (w/v) of respective substrate. Chitosan (deacetylated chitin from crab shells, Wako Ind. Ltd, Osaka, Japan), chitin (from crab shells, Wako Ind. Ltd, Osaka, Japan), curdlan (Wako Ind. Ltd, Osaka, Japan), laminarin (from *Laminaria digitata*, Sigma-Aldrich, St. Louis, USA) and glucan from black yeast (yeast glucan) (Tokyo Chemical Industry Co. Ltd, Tokyo, Japan) were used as polysaccharide substitutes to the components of the ascomycetes fungal cell wall. Chitosan and chitin are polymers of glucosamine and *N*-acetylglucosamine (GlcNAc), respectively, curdlan is a linear polymer of β-1,3-glucan, and laminarin is composed of both β-1,3-glucan and β-1,6-glucan. Laminarin was soluble in the water. However, chitin, chitosan, curdlan, and yeast glucan were insoluble. In autoclaved PY broth supplemented with these substrates (0.5%, w/v), the first two compounds (chitin and chitosan) produced a translucent hydrogel, curdlan precipitated as a lumpy material of hydrogel, whereas yeast glucan diffused as cloudy precipitates. Therefore, they were used as insoluble substrates without any further treatment. Baker’s dry yeast (freeze-dried cells of the ascomycetes yeast *Saccharomyces cerevisiae,* Nisshin Food Products Co. Ltd., Tokyo, Japan), wheat bran, and dried leaves of *B. juncea* were also used as substrates for the cultivation of strains H110 and TB8 to examine production of the enzymes. Dried leaves of *B. juncea* were ground into coarse powder using a mortar and pestle for the ease of medium preparation. Each of the three substrates was added to the PY broth (1%, w/v) and then the medium was autoclaved, in which these substrates precipitated as solid sediment. All the cultivation experiments of both the strains were carried out at 30 °C, unless otherwise stated.

The substrate utilization ability for each substrate was determined by monitoring the increase in culture turbidity (OD_660_) as well as the quantity of the products as compared to those of the cultures cultivated without substrates (PY broth only). Especially, the abilities to degrade insoluble substrates were determined by measuring fermentation products in the culture supernatants after the cultivation. Fermentation products, such as volatile fatty acids (VFAs), alcohols, and gases (H_2_ and CO_2_), were analyzed by gas chromatography (GC) (Hitachi G-3000 or G-5000, Tokyo, Japan) as described previously (Ueki et al. 2009, 2016). Glass columns (0.3 cm × 200 cm) packed with 20% polyethylene glycol adipate (EGA) and 2% H_3_PO_4_ coated on Chromosorb AW (60/80 mesh) (for VFAs) and PEG1000 on Uniport B (60/80 mesh) (for alcohols) were used for analyses using GC equipped with a flame ionization detector (FID). Gases were analyzed with a metal column packed with activated carbon (30/60 mesh) by thermal conductivity detector (TCD). All the experiments for measurement of the products were performed in duplicates.

### Determination of β-1,3-glucanase and chitosanase activities

β-1,3-Glucanase and chitosanase activities of both the strains (H110 and TB8) were determined using supernatants of cultures supplemented with different substrates. After cultivation in the PY broth containing respective substrate as shown below, the culture supernatants were collected by centrifugation at 25,000×*g* for 20 min and were stored at − 20 °C until use. β-1,3-Glucanase activity was assessed with laminarin (Sigma-Aldrich, St. Louis, USA) as a water-soluble substrate according to the standard method (Aktuganov et al. 2007; Zacky and Ting 2013). The reaction mixture was composed of 1 ml of culture supernatant as an enzyme solution, 0.5 ml of laminarin solution (0.5%, w/v), and 1 ml of 10 mM potassium phosphate buffer (pH 7.0). The culture supernatants as the enzyme solutions were diluted appropriately, when required. The reaction mixture was incubated for 15 and 30 min at 38 °C in a water bath and was stopped by transferring in ice-cold water. Reducing sugar in the reaction mixture was measured by 3,5-dinitrosalicylic acid (DNS) method (Miller 1959) at 535 nm based on the standard curve prepared by using different concentrations of glucose. One unit of enzyme activity was defined as the amount of enzyme catalyzing the formation of 1 μmol reducing sugar (as glucose equivalent) in 60 min under conditions described above.

Chitosanase activity was determined according to the method described previously (Imoto and Yagishita 1971; Saito et al. 2009). The composition of the reaction mixture for checking chitosanase activity was 1 ml of 0.25% (w/v) chitosan (Sigma-Aldrich, St. Louis, USA) solution in 100 mM acetate buffer (pH 5.3; to solubilize chitosan at low pH), and 1 ml of the enzyme solution (culture supernatant). The reaction mixture was incubated at 38 °C for 15 and 30 min in a water bath and was stopped by transferring in ice-cold water. Reducing sugar was determined by the DNS method. One unit of the enzyme activity was defined as the amount of enzyme catalyzing the formation of 1 μmol reducing sugar (as glucosamine equivalent determined based on the standard solutions of glucosamine) in 60 min under the conditions similar to that of the β-1,3-glucanase activity. Both the enzyme assays were performed at least in four replicates.

### Effects of the growth of the anaerobic bacteria on the survival of the *Fusarium* pathogen under anaerobic condition

Effects of the growth of the anaerobic bacterial strains (H110 and TB8) on the survival of the fungal pathogen were examined by co-incubation of each strain with the pathogen in the PY broth containing glucose (PYG) or wheat bran. The nitrate-non-utilizing (*nit*) mutant of *F. oxysporum* f. sp. *spinaciae* strain M2-1 (= MAFF150001) (Mowlick et al. 2013a; Takehara et al. 2003; Ueki et al. 2017) was used as the wilt pathogen of spinach. Strain M2-1 was streaked on potato dextrose agar (PDA) plates and cultivated for 7 days at 28 °C. The PDA medium with densely overgrown mycelia was cut into square pieces (approximately 6 mm × 6 mm). Three such pieces were placed into the respective medium (PY broth containing glucose or wheat bran) (10 ml) prepared in test tubes for each anaerobic bacterial strain (20–25 replicates). Each bacterial strain (0.1 ml of 24 h old culture cultivated in the PYG broth) was inoculated into the medium containing the agar pieces and was incubated anaerobically at 25 °C (PYG) or 30 °C (PY broth with wheat bran). Simultaneously, replicates of the control culture without inoculation of the anaerobic bacterial strains (i.e., the *Fusarium* pathogen alone) were also incubated. Agar pieces were taken out from four or five culture tubes randomly on each sampling day during the incubation period of 3 weeks and were cut into smaller pieces (approximately 2 mm × 6 mm), which were immediately placed onto two fresh PDA plates (four or five pieces per plate). The agar plates were incubated at 28 °C to examine mycelial growth of the pathogen from each agar section. Based on the microscopic examination of the mycelial cells on the agar pieces, more than 1 × 10^4^ cells were present on each agar section placed on the PDA plates (Ueki et al. 2017). The remaining agar sections were stored in 5% (v/v) formaldehyde solution to observe the fungal cells by microscopy.

### Preparation of freeze-dried whole-cell biomass of *Fusarium oxysporum* f. sp. *spinaciae* as a substrate for cultivation of strains H110 and TB8

Freeze-dried whole-cell fungal biomass to examine the ability of strains H110 and TB8 to degrade it as a substrate for growth was prepared by following method. The *Fusarium* pathogen strain M2-1 was cultivated in the potato dextrose (PD) broth for 7 days at 28 °C by using a reciprocal shaker (100 rpm). The fungal biomass was harvested by centrifugation at 10,000×*g* for 20 min, which was then resuspended in distilled water and washed again by centrifugation at 10,000×*g* for 20 min. The resulting pellet was freeze-dried and stored at 4 °C. The freeze-dried mycelial cells were added to the PY broth (1%, w/v), which was then autoclaved.

### Time-dependent degradation of mycelial cells and changes in enzyme activities during cultivation of strains H110 and TB8 with the freeze-dried biomass of *F. oxysporum* f. sp. *spinaciae* strain M2-1

To examine the time courses of enzyme activities of the H110 and TB8 cultures containing freeze-dried biomass of strain M2-1 as a substrate, culture tubes of the PY broth (10 ml) containing the biomass were prepared. Each strain (H110 or TB8) cultivated in the PYG broth for 24 h was inoculated into 20–25 replicates of tubes as described above. Four or five culture tubes were picked up randomly on each sampling day during their incubation period of 3 weeks. The culture supernatants were harvested and mycelial cell samples were kept in 5% (v/v) formaldehyde solution. β-1,3-Glucanase and chitosanase activities in the obtained culture supernatants were determined and mycelial cells were observed by fluorescence microscopy. VFAs and alcohols produced in the culture supernatants were also determined as described above.

### Observation of *F. oxysporum* f. sp. *spinaciae* strain M2-1 cells by fluorescence microscopy

After the anaerobic incubation of the mycelial cells of *F. oxysporum* f. sp. *spinaciae* strain M2-1 (live cells on PDA sections or freeze-dried dead cells), the cells were harvested from the medium and stored in 5% (v/v) formaldehyde solution as described above. For fluorescence microscopy, calcofluor white stain (Sigma-Aldrich Co. LLC, St. Louis, USA) was used to observe the mycelial cells according to the manufacturer’s instructions. A fluorescence microscope (Olympus BX51, Olympus Corp. Tokyo, Japan) equipped with Million Pixel Camera System (Carl Zeiss, Oberkochen, Germany) was used.

### Accession numbers of the sequences

Nucleotide sequences of 16S rRNA gene of strains H110 and TB8 were deposited in DDBJ/GenBank under the accession numbers LC020492 and LC020493, respectively.

## Results

### β-1,3-Glucanase and chitosanase activities in the culture supernatants of strains H110 and TB8 cultivated with different substrates

Strains H110 and TB8 were cultivated with different carbohydrates for 7 days and the culture supernatants were harvested to determine the concentrations of the products as well as the β-1,3-glucanase and chitosanase activities. Both the strains degraded different types of glucan (curdlan, laminarin, and yeast glucan) as well as chitosan. Growth of both the strains with the soluble substrates (glucose and laminarin) was confirmed by the increase in the culture turbidity (OD_660_) and the quantities of the products (acetate, butyrate, H_2_, and CO_2_). Ethanol and *n*-butanol were also produced from glucose by both the strains (data not shown). Table 1 shows the products of the insoluble substrates (curdlan, yeast glucan, and chitosan) and the PY broth without substrate supplement. Minor amounts of acetate and butyrate were produced from the PY broth, whereas much higher amounts of butyrate were produced from the polysaccharides by both the strains. Substantial amounts of *n*-butanol were produced only from curdlan.

**Table 1:** Products in the culture supernatants used for determination of enzymatic activities for strains H110 and TB8

Strain	Substrate	Products in the culture supernatant (mmol/l)			
		Acetate	Butyrate	Ethanol	*n*-Butanol
H110	None (PY)	0.73 ± 0.09	3.60 ± 0.05	1.63 ± 0.05	–
	Curdlan	4.94 ± 0.58	15.9 ± 2.11	0.78 ± 0.06	14.9 ± 0.13
	Yeast glucan^a^	0.42 ± 0.01	12.7 ± 0.45	1.45 ± 0.05	1.42 ± 0.07
	Chitosan	4.89 ± 0.22	19.5 ± 0.21	0.72 ± 0.07	–
TB8	None (PY)	0.92 ± 0.05	2.90 ± 0.01	0.85 ± 0.04	–
	Curdlan	4.28 ± 0.01	22.0 ± 0.49	–	5.68 ± 0.52
	Yeast glucan^a^	0.87 ± 0.04	14.0 ± 0.85	1.08 ± 0.07	0.62 ± 0.09
	Chitosan	4.55 ± 0.20	24.7 ± 0.82	0.70 ± 0.24	–

Concentrations of substrate, 0.5% (w/v). Both the strains produced H_2_ and CO_2_ from all substrates

Cultivated for 7 days for all substrates and the supernatant of each culture was used for determination of enzymatic activities shown in Table 2

All data are mean values (± SD) of duplicate experiments

*–* not detected

^a^Glucan from black yeast (Tokyo Chemical Industry Co., Ltd)

Table 2 shows β-1,3-glucanase and chitosanase activities in the supernatants of both the strains cultivated with these substrates. Strain H110 showed almost same level of β-1,3-glucanase activity in all the three different types of glucans, whereas TB8 showed higher activity with laminarin than that with curdlan and yeast glucan. When cultivated with chitosan or glucose, high β-1,3-glucanase activity was detected from both the strains. Both the strains showed high chitosanase activity when cultivated with chitosan. The activity was also detected with both curdlan and glucose; however, it was weak for both the strains when grown with curdlan.

**Table 2:** β-1,3-Glucanase and chitosanase activities of strains H110 and TB8 grown with various saccharides in the PY broth

Strain	Substrate	Activities (Unit/ml)	
		β-1,3-Glucanase	Chitosanase
H110	None (PY)	0.21 ± 0.02	–
	Curdlan	5.10 ± 0.43	1.43 ± 0.21
	Laminarin	6.60 ± 0.13	nd
	Yeast glucan^a^	5.16 ± 1.05	nd
	Chitosan	3.47 ± 0.33	5.58 ± 1.32
	Glucose	3.78 ± 0.18	5.00 ± 0.69
TB8	None (PY)	0.14 ± 0.03	–
	Curdlan	2.47 ± 1.48	0.26 ± 0.06
	Laminarin	5.19 ± 0.18	nd
	Yeast glucan^a^	2.45 ± 0.29	nd
	Chitosan	2.31 ± 0.03	4.26 ± 0.34
	Glucose	3.30 ± 0.91	3.41 ± 0.26

Activities, mean ± SD (n = 4)

Cultivated for 7 days for all substrates

*–* not detected, *nd* not determined

^a^Glucan from black yeast (Tokyo Chemical Industry Co., Ltd)

### Co-incubation of live cells of *F. oxysporum* f. sp. *spinaciae* strain M2-1 with strains H110 or TB8 in the PYG broth

Strains H110 and TB8 secreted both β-1,3-glucanase and chitosanase enzymes in the culture supernatants when cultivated in the PYG broth as shown above (Table 2). Thus, live cells of the *Fusarium* pathogen strain M2-1 grown on PDA pieces were co-incubated with H110 and TB8 at 25 °C in the PYG broth to investigate whether these bacterial strains have the ability to disrupt live mycelial cells thereby killing the pathogen. Though the growth rates of both the strains at the exponential growth phase were slightly lower at 25 °C than that at 30 °C, both grew rapidly in the PYG broth and produced almost same amounts of fermentation products as those produced at 30 °C (data not shown).

**Fig. 1:**
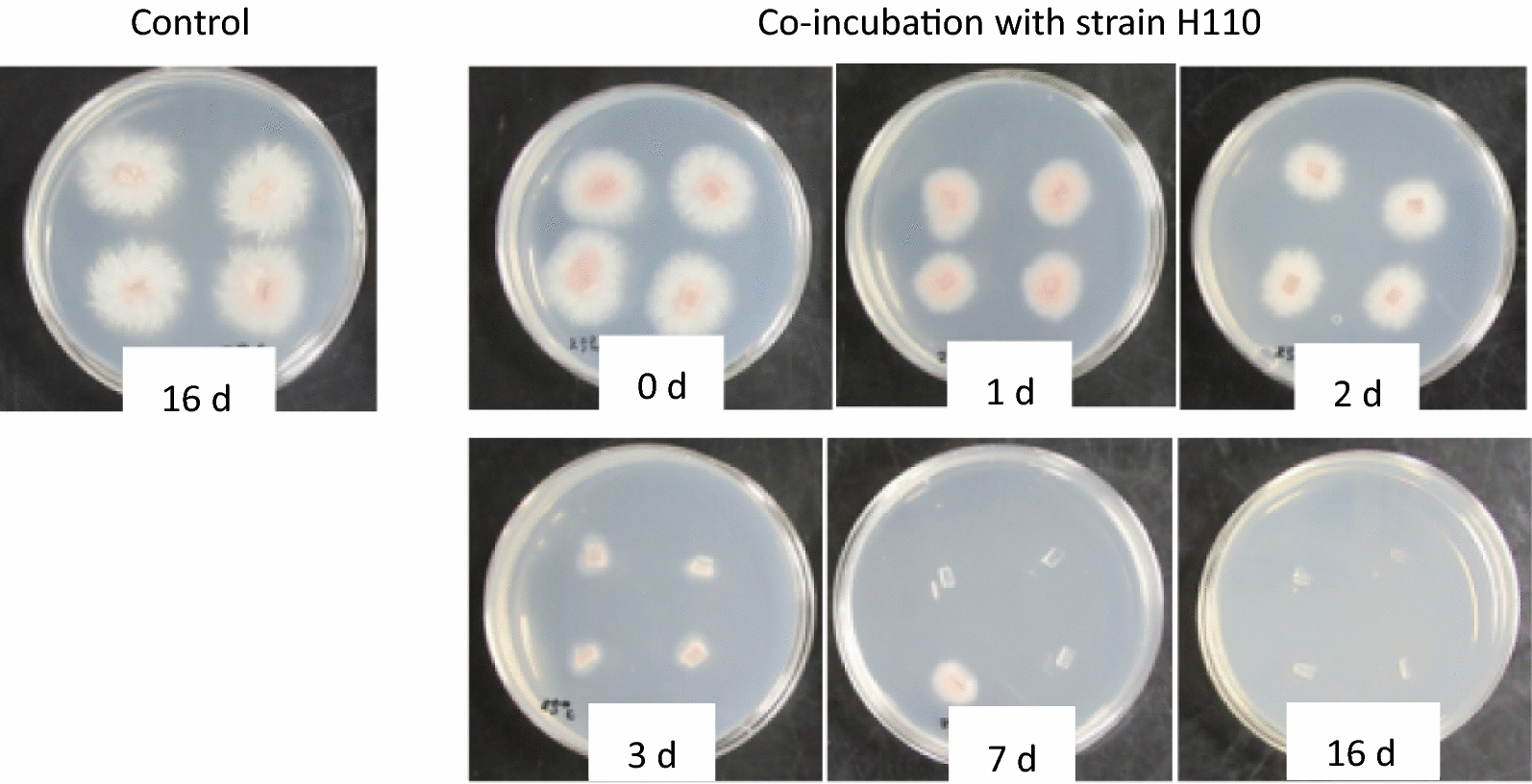
Growth of mycelium of *Fusarium oxysporum* f. sp. *spinaciae* strain M2-1 from agar sections incubated alone without strain H110 (control, for 16 days) or co-incubated with strain H110 (for 0, 1, 2, 3, 7, 16 days) in the PYG broth at 25 °C. The photos were taken 2 days (agar sections co-incubated for 0, 1, 2, 3 days) or 6 days (co-incubated for 7, 16 days) after incubation of the agar sections on the fresh PDA plates at 28 °C. For the control sample, the photo was taken after 3 days incubation of the PDA plate. For each sampling day, 2 fresh PDA plates (4 agar sections per plate) were used to check growth of the pathogen from the agar sections

The fungal pathogen incubated alone (control incubation) and that recovered from the PYG broth rapidly developed the mycelium from the agar sections when placed on the fresh PDA plates irrespective of incubation periods, indicating that the *Fusarium* cells were alive in PYG even after 16 days of the strict anaerobic incubation (Fig. 1). In contrast, growth of the *Fusarium* mycelium co-incubated for 3 days with strain H110 was significantly delayed and the fungal growth from the co-incubated agar sections was distinctly suppressed in accordance with the length of co-incubation periods (Fig. 1). Development of the mycelium from the 7 days old co-incubated agar sections was late and weak and observed only in two out of eight agar sections placed on the PDA plates. After 16 days of co-incubation with strain H110, none of the agar sections developed the mycelium, indicating that all the fungal cells on the agar sections died during the co-incubation. Similarly, when co-incubated for 16 days with strain TB8 under the same conditions, all the pathogenic fungal cells on the agar sections were dead, though the fatal effects on the pathogen in the early periods of the co-incubation seemed to be weaker than that by strain H110.

### Observation of the *Fusarium* mycelial cells co-incubated with the anaerobic bacteria by fluorescence microscopy

**Fig. 2:**
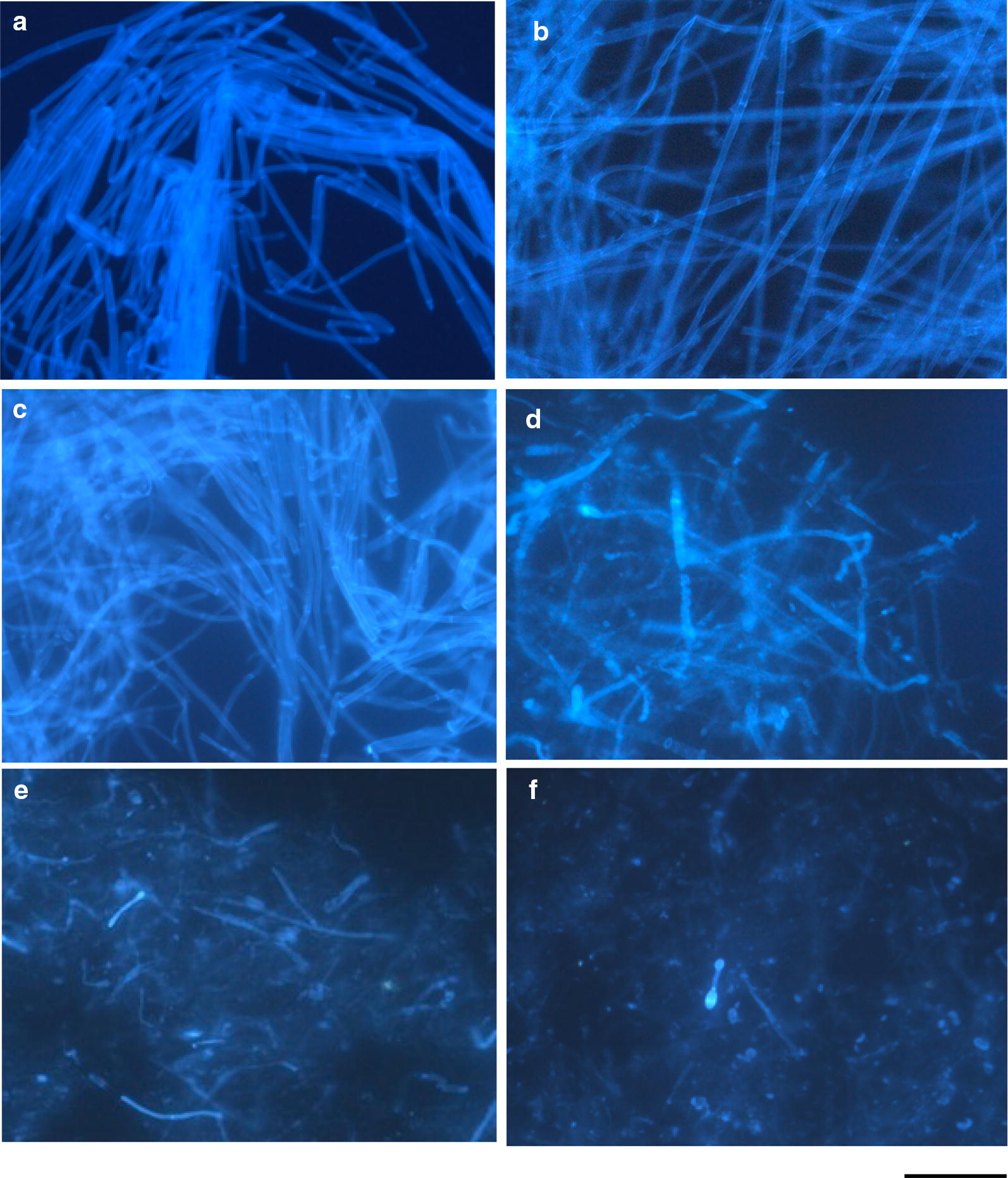
Fluorescence photomicrographs of mycelial cells of *F. oxysporum* f. sp. *spinaciae* strain M2-1 anaerobically incubated in the PYG broth at 25 °C. **a** Intact mycelial cells before the start of the incubation; **b** after the control incubation of the pathogen alone (without strain H110) for 16 days; **c**–**f** co-incubated with strain H110 for 1 day (**c**), 2 days (**d**), 3 days (**e**), and 7 days (**f**). Live mycelial cells of strain M2-1 on agar sections of PDA were inoculated into the PYG broth and the agar sections taken out from the culture tubes on each sampling day were used for the microscopic observation. Bar, 30 μm

*Fusarium* mycelial cells co-incubated anaerobically in the PYG broth with H110 and TB8 were observed by fluorescence microscopy using calcofluor white as a fluorescent dye. The intact mycelial cells before the start of the co-incubation emitted light blue fluorescence from whole cells showing clear septa with stronger luminous intensity (Fig. 2a). Similar fluorescence micrographs were obtained for the mycelial cells incubated anaerobically alone in the PYG broth for 16 days (Fig. 2b), suggesting that the anaerobic condition did not cause any significant damages to the fungal cells. Figure 2c–f shows the micrographs of the *Fusarium* cells co-incubated with strain H110. The fungal cells co-incubated for 1 day emitted similar fluorescence as that of the cells before the co-incubation, suggesting that the cells were not damaged during the first day of co-incubation. However, severe disruption of the cells was obvious in the cells co-incubated for more than 2 days. Very few undamaged cells (or thick-walled chlamydospore-like cells) with strong luminous intensity were distinguished in the agar sections co-incubated for more than 3 days. The cells that survived barely should develop delayed mycelial colonies as shown in Fig. 1. Observation by fluorescence microscopy revealed that strain TB8 also destroyed the co-incubated fungal cells in a similar manner as that of strain H110, though the disruption process was slower than that by strain H110 (data not shown).

### Cultivation of strains H110 and TB8 with freeze-dried dead *Fusarium* biomass and dry yeast as growth substrates

To study the ability of strains H110 and TB8 to directly utilize or decompose fungal mycelial biomass as a growth substrate, both the strains were cultivated with the freeze-dried dead whole-cell biomass of *F. oxysporum* f. sp. *spinaciae* strain M2-1 in the PY broth. Baker’s dry yeast, freeze-dried cells of the ascomycetes yeast *S. cerevisiae,* was also examined. Since the PY broth supplemented with these fungal biomass was autoclaved, all the fungal cells were dead. Table 3 shows the amounts of products and the enzyme activities in the supernatant of 7 days old culture of both the substrates. Both strains produced high amount of butyrate as a major product with smaller amounts of acetate and alcohols from both substrates, indicating that both the strains utilized both biomass samples as growth substrates. Both β-1,3-glucanase and chitosanase activities were detected in the culture supernatants of both the strains, though the β-1,3-glucanase activity of strain TB8 was weaker than that of strain H110 for both fungal biomass.

**Table 3:** Products and enzymatic activities in the culture supernatants of strains H110 and TB8 grown with cell biomass of the *Fusarium* pathogen and yeast in the PY broth

Strain	Substrate	Products in the culture supernatant (mmol/l)				Activities (Unit/ml)	
		Acetate	Butyrate	Ethanol	*n*-Butanol	β-1,3-Glucanase	Chitosanase
H110	*Fusarium* biomass	3.25 ± 0.22	15.1 ± 0.75	1.26 ± 0.29	0.86 ± 0.08	4.02 ± 0.53	3.19 ± 1.05
	Dry yeast	1.44 ± 0.03	12.7 ± 0.55	–	1.69 ± 0.25	3.65 ± 0.05	3.40 ± 0.58
TB8	*Fusarium* biomass	1.05 ± 0.01	9.4 ± 0.17	–	0.31 ± 0.15	1.03 ± 0.03	1.01 ± 0.06
	Dry yeast	0.78 ± 0.10	13.4 ± 0.20	–	1.55 ± 0.59	1.11 ± 0.77	3.88 ± 0.10

*Fusarium* biomass, freeze-dried and killed mycelial cells of *Fusarium oxysporum* f. sp. *spinaciae* M2-1

Concentration of substrates, 1% (w/v). Products, mean ± SD (n = 2); activities, mean ± SD (n = 4)

Cultivated for 7 days for both the substrates

*–* not detected

### Time courses study of β-1,3-glucanase and chitosanase activities during cultivation of H110 and TB8 with the freeze-dried *Fusarium* biomass

**Fig. 3:**
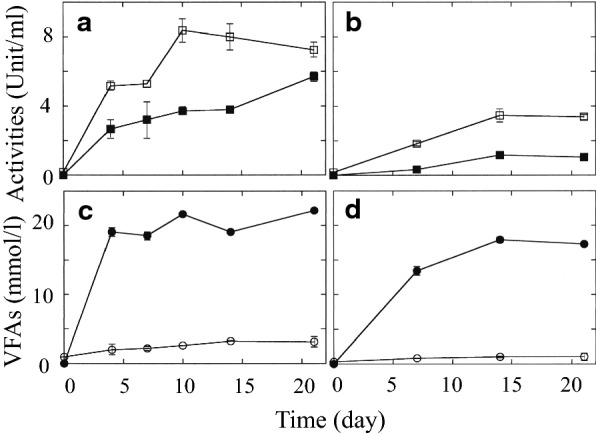
Time courses of enzymatic activities and production of volatile fatty acids (VFAs) for strains H110 (**a**, **c**) and TB8 (**b**, **d**) with the freeze-dried dead biomass of *F. oxysporum* f. sp. *spinaciae* strain M2-1 as a substrate (1%, w/v) in the PY broth. open square, β,1-3-glucanase; filled square, chitosanase; open circle, acetate; filled circle, butyrate. Strain H110 produced small amounts of ethanol (< 1 mmol/l) and *n*-butanol (< 2 mmol/l). Strain TB8 produced none of alcohols. Error bars represent the standard deviations

Strains H110 and TB8 were cultivated for 3 weeks at 30 °C in the PY medium containing the freeze-dried dead biomass of the *Fusarium* pathogen strain M2-1. Figure 3a shows the changes in the β-1,3-glucanase and chitosanase activities of strain H110 during cultivation. The β-1,3-glucanase activity was detected in the culture supernatant of day 4, which was retained until the end of cultivation for 3 weeks. In case of the chitosanase activity, it was lower than that of β-1,3-glucanase, and was detected throughout the cultivation period. The result showed that these enzyme activities are stable in the anaerobic conditions at 30 °C for at least 3 weeks. Figure 3c shows that strain H110 produced mainly butyrate, and the concentrations of the products reached the plateau by fourth day of cultivation. Minor amounts of ethanol and *n*-butanol were also detected in the culture supernatants.

The enzyme activities of strain TB8 were low at day 7, however, the activities increased by day 14, which was retained till the end of incubation (3 weeks) (Fig. 3b). Strain TB8 also produced butyrate as the major product, and the time-dependent increase in the concentration was almost in parallel with the increase in the enzyme activities (Fig. 3d).

### Observation of the freeze-dried mycelial cells of the *Fusarium* spinach wilt pathogen by fluorescence microscopy

**Fig. 4:**
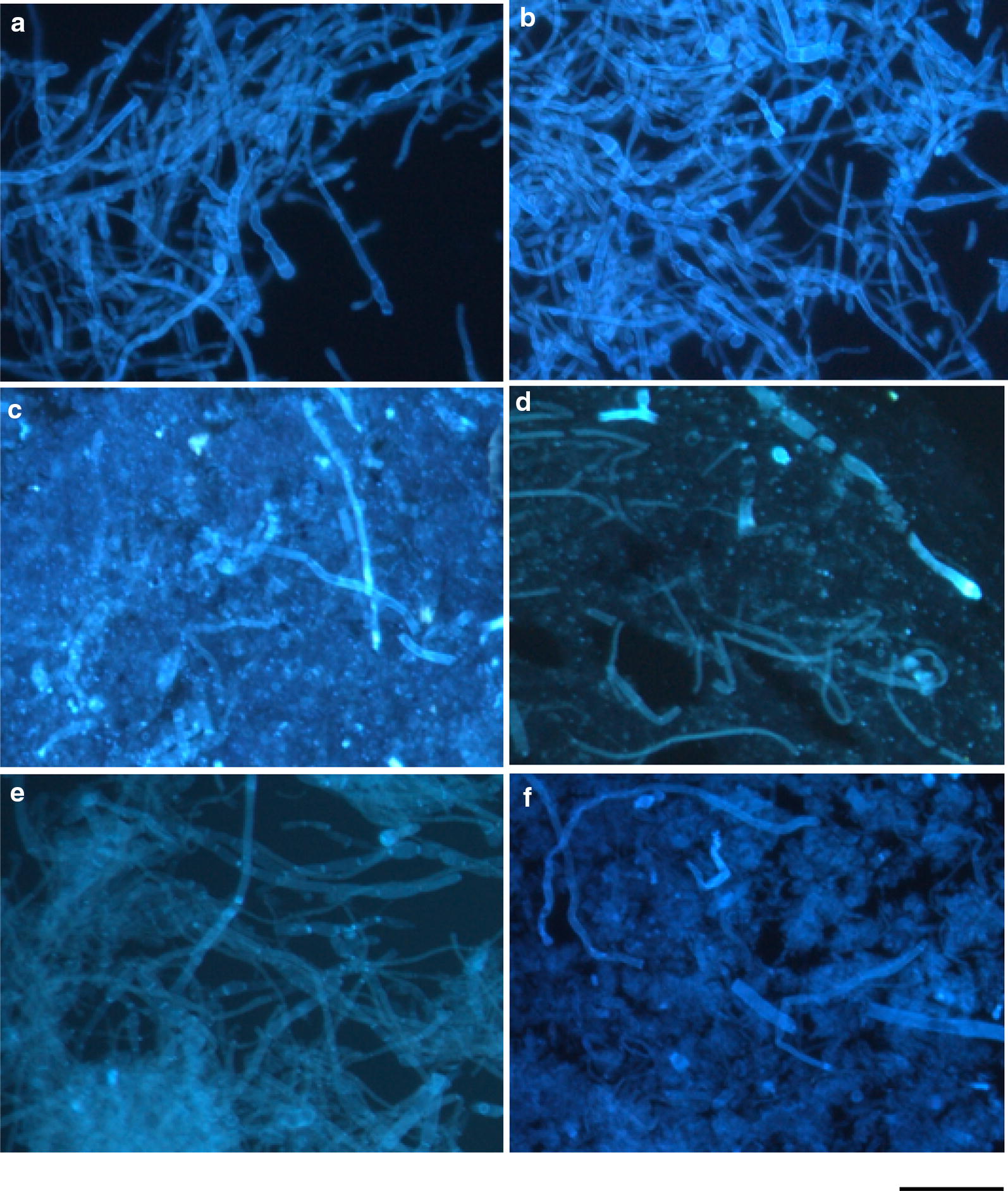
Fluorescence photomicrographs of the freeze-dried dead biomass of *F. oxysporum* f. sp. *spinaciae* strain M2-1 incubated under the anaerobic conditions in the PY broth. **a** At the start of incubation; **b** after the anaerobic incubation of the pathogen biomass alone for 21 days; **c**, **d** incubated with strain H110 for 4 days (**c**) and 7 days (**d**); **e**, **f** incubated with strain TB8 for 7 days (**e**) and 14 days (**f**). Bar, 30 μm

The mycelial cells of the freeze-dried dead biomass of strain M2-1 after the incubation were observed by fluorescence microscopy. Figure 4a shows the fluorescence micrograph of the freeze-dried cells at the start of incubation. The freeze-dried and autoclaved mycelial cells emitted similar fluorescence as shown by the intact living cells (Fig. 2a) and similar micrographs were obtained from the mycelia incubated alone anaerobically for 21 days. Figure 4c–f shows the micrographs of the freeze-dried *Fusarium* cells incubated with each anaerobic bacterial strain. The fungal cells were notably disrupted into small pieces by fourth day of incubation with strain H110 (Fig. 4c). When observing the fungal cells incubated with strain H110 for 7 days, it was difficult to confirm their presence because of the excessive destruction. The *Fusarium* cells incubated for 7 days with strain TB8 did not show significant destruction (Fig. 4e), whereas degradation of the fungal cells was evident after 14 days of incubation (Fig. 4f). The time courses study of the destruction of mycelia observed by fluorescence microscopy for strain TB8 corresponded well with the increase in the enzyme activities shown in Fig. 3b.

### Effects of strains H110 and TB8 growth using wheat bran as a substrate on live *Fusarium* cells during co-incubation

**Fig. 5:**
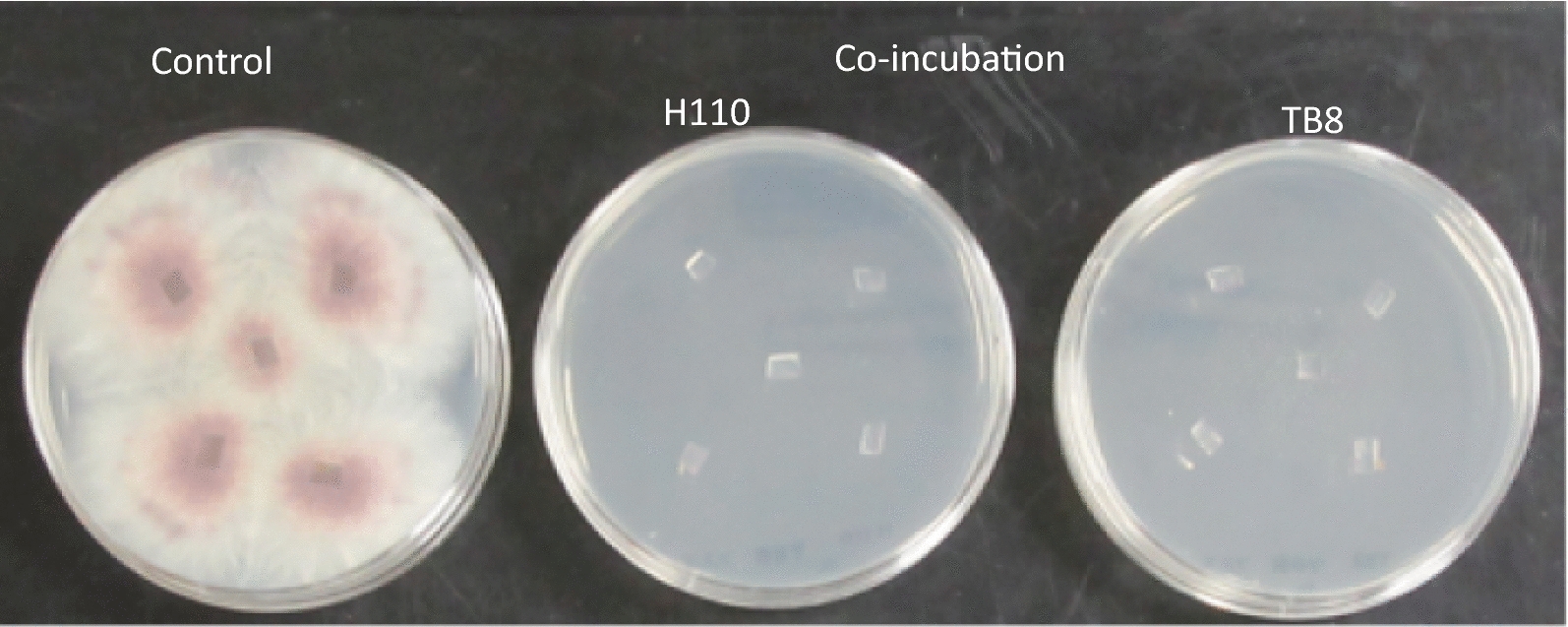
Growth of mycelium of *Fusarium oxysporum* f. sp. *spinaciae* strain M2-1 from agar sections incubated alone without anaerobic bacterial strains (control) or co-incubated with strains H110 or TB8 for 7 days at 30 °C in the PY broth containing wheat bran (1%, w/v) as a substrate. The photos were taken after 6 days incubation of the PDA plates at 28 °C. Two fresh PDA plates (5 agar sections per plate) were used to check growth of the pathogen from the agar sections for each sample

Live cells of the *Fusarium* pathogen strain M2-1 on PDA pieces were co-incubated in the anaerobic medium with each strain using wheat bran as a material for the BSD treatment. Figure 5 shows the PDA plates with agar sections co-incubated for 7 days with each anaerobic strain and then incubated for 6 days after transfer on the fresh PDA plates. The mycelium of the pathogen incubated alone (control) grew normally from the agar sections, whereas none of the agar sections incubated with each anaerobic strain showed extension of the mycelium, suggesting that both H110 and TB8 inactivated or killed all the cells of the pathogen on the PDA sections during the anaerobic co-incubation with wheat bran. The pathogen incubated alone for 3 weeks under the same conditions also rapidly developed mycelia after placement on the PDA plates without any inactivation of the cells. When the mycelial cells of the *Fusarium* pathogen strain M2-1 on the agar pieces co-incubated anaerobically with each strain using wheat bran were observed by fluorescence microscopy, similar degradation of the fungal cells was observed as that with strain H110 in PYG medium (Fig. 2). However, strain TB8 degraded the fungal cells at the earlier period of the co-incubation than that in the PYG broth (data not shown).

### Enzymatic activities of the strains H110 and TB8 in the culture supernatant with wheat bran and *Brassica* plant biomass as substrate

The fermentation products and enzymatic activities in the supernatants for 7 days co-incubated culture with wheat bran shown above were determined (Table 4). Both strains produced similar amounts of acetate and butyrate as those from curdlan (Table 1). High β-1,3-glucanase activities were detected for both strains, especially for strain TB8. Chitosanase activities were also detected, but the activities were lower as compared with the levels of β-1,3-glucanase activity for both strains. The high β-1,3-glucanase activity for strain TB8 corresponded well with the early death of the *Fusarium* pathogen during the co-incubation with the strain in wheat bran.

**Table 4:** Products and enzymatic activities in the culture supernatants of strains H110 and TB8 grown with wheat bran and *Brassica* leaves in the PY broth

Strain	Substrate	Days of cultivation	Products in the culture supernatant (mmol/l)		Activities (Unit/ml)	
			Acetate	Butyrate	β-1,3-Glucanase	Chitosanase
H110	Wheat bran^a^	7	1.24 ± 0.05	16.2 ± 0.43	4.92 ± 0.12	1.50 ± 0.08
	Wheat bran	21	2.16 ± 0.31	17.2 ± 0.61	3.70 ± 0.51	1.10 ± 0.19
	Brassica leaf	21	2.51 ± 0.82	7.41 ± 0.12	1.47 ± 0.27	nd
TB8	Wheat bran^a^	7	0.99 ± 0.03	16.5 ± 0.45	8.25 ± 0.02	1.32 ± 0.05
	Wheat bran	21	2.10 ± 0.27	15.0 ± 0.38	6.67 ± 0.05	1.72 ± 0.06
	Brassica leaf	21	1.60 ± 0.31	5.94 ± 0.06	1.51 ± 0.47	nd

Products, mean ± SD (*n* = 2); activities, mean ± SD (*n* = 4)

Both strains produced small amounts (< 2 mmol/l) of *n*-butanol

*nd* not determined

^a^Co-incubated with *F. oxsporum* f. sp. *spinaciae* strain M2-1. Concentrations of substrates, 1% (w/v)

When both the strains were cultivated alone with wheat bran for 3 weeks, almost similar levels of fermentation products and enzyme activities were detected as those from the cultures co-incubated for 7 days (Table 4). The *Brassica* plant biomass was also examined by cultivating both strains alone for 3 weeks. Both the strains produced lower amounts of acetate and butyrate as compared with those from wheat bran. Although the enzyme activities were lower than those with wheat bran, β-1,3-glucanase was detected in both strains. Chitosanase activities were not determined.

## Discussion

The mechanisms behind suppression of soil-borne plant pathogens by the BSD-treatments have been investigated extensively from various viewpoints (Blok et al. 2000; Momma 2008; Strauss and Kluepfel 2015). In the previous study (Ueki et al. 2017), we examined the contribution of anaerobic bacteria into the suppression of plant pathogens by using anaerobic bacterial strains (H110 and TB8) isolated from the soil samples subjected to the BSD-treatments. Both the bacterial strains grew rapidly at 30 °C in the strictly anaerobic conditions by using various carbohydrate substrates including polysaccharides composing ascomycetes fungal cell wall. Both strains killed the spinach wilt disease pathogen (*F. oxysporum* f. sp. *spinaciae* strain M2-1) when co-incubated under the anaerobic conditions. Significant destruction of the fungal mycelial cells was observed by phase-contrast microscopy. In this study, production of the enzymes by both the strains to degrade polysaccharides related to the ascomycetes fungal cell wall was investigated to further clarify the functions of the anaerobic bacteria in the BSD-treatments. Furthermore, fungal cells were observed by fluorescence microscopy to examine the degradation process of fungal cell wall more definitely.

The compositions of glucan used in this study as substrates for cultivation of both anaerobic strains are different from each other. Curdlan is a linear polymer of β-1,3-glucan. The major component in the backbone of laminarin is also β-1,3-glucan, however, it contains a small amount of consecutive β-1,6-linked glycosides in the branched chains. The ratio of the 1,3 to 1,6 bonds in laminarin from *L. digitata* has been estimated to be 9.51:1 (Liu et al. 2018). The composition of the commercially available reagent ‘yeast glucan’ used in this study has not been presented. Although the ‘yeast glucan’ seemed to be inferior to other two types of glucans as a substrate of the growth, all of them supported rapid growth and fermentation of strains H110 and TB8, indicating that both the strains have the ability to easily decompose these polysaccharides immediately after inoculation into the medium. Determination of enzyme activities confirmed the production of extracellular β-1,3-glucanase in the presence of all these different types of glucans by both the strains. Production of extracellular chitosanase was also confirmed in the culture supernatants with chitosan. High β-1,3-glucanase activities were detected in the culture supernatants using chitosan or glucose and chitosanase activity in the culture with glucose or curdlan, suggesting that strains H110 and TB8 might produce both enzymes in accordance with the growth regardless of the substrates available in the medium. Production of β-1,3-glucanase and chitosanase exhibiting anti-fungal activities has been reported in some aerobic bacteria (Aktuganov et al. 2007; Kurakake et al. 2013; Prasanna et al. 2008) and also in some plants (Cota et al. 2007; Egea et al. 1999) and fungi (Masih and Paul 2002; Ruiz-Dueñas and Martínez 1996). A few reports on the production of β-1,3-glucanase by obligate anaerobic bacteria have been reported (Dvortsov et al. 2009), whereas the ability of *C. beijerinckii* to degrade fungal-cell-wall polysaccharides has not been reported.

When we co-incubated strain H110 or TB8 with the live *Fusarium* pathogen strain M2-1 in the PYG medium at 30 °C under the anaerobic culture conditions in our previous study (Ueki et al. 2017), both strains completely killed the pathogen during the co-incubation of 3 weeks. Conspicuous degradation of the fungal cells observed by phase-contrast microscopy suggested production of extracellular cell-wall degrading enzymes by the anaerobic bacteria during the co-incubation in the PYG broth. Detection of β-1,3-glucanase and chitosanase activities in the culture supernatants of the PYG for both the strains in this study confirmed the results obtained in the previous study (Ueki et al. 2017). In this study, the co-incubation of the *Fusarium* pathogen strain M2-1 with each of anaerobic bacterial strains in the PYG broth was conducted at 25 °C, because the anaerobic bacteria destroyed the fungal cells so rapidly at the initial period of the co-incubation at 30 °C that the detailed observation of the early process of fungal cell destruction was difficult. The results of co-incubation at 25 °C obtained in this study showed that the pathogen was intensely attacked by the anaerobic bacteria in accordance with the bacterial growth during the first 2 days of co-incubation. It was also shown that both the strains have potential to kill the *Fusarium* pathogen at 25 °C as well as at 30 °C.

The *Fusarium* pathogen strain M2-1 used in this study is an ascomycetes fungus. The ascomycetes fungal cell wall generally has an inner layer composed of a cross-linked chitin-glucan matrix (Arroyo et al. 2016; Horiuchi 2009). Among the polysaccharides present in the fungal cell wall, β-1,3-glucan is the most abundant (70–80%) and β-1,6-glucan and chitin comprise less than 10%, respectively (Arroyo et al. 2016; Geoghegan et al. 2017; Ruiz-Herrera and Ortiz-Castellanos 2010; Schoffelmeer et al. 1999). Though chitin (a polymer of GlcNAc) is generally rich in the inner layer of the cell wall of the most fungi (Munro and Gow 2001), it is a major component of the fungal septum wall (Horiuchi 2009; Hunsley and Gooday 1974; Mouriño-Pérez 2013). Chitosan, another important component of fungal cell wall, is usually a heterogeneous polymer of GlcNAc and glucosamine. Chitosan is synthesized through the deacetylation of GlcNAc in the chitin chains by specific deacetylases (Geoghegan and Gurr 2017; Ruiz-Herrera and Ortiz-Castellanos 2010). Fukamizo et al. (1996) reported that the degree of acetylation of the chitinous component in the cell wall of *F. oxysporum* is estimated to be 25–35% of the chitinous chains (approximately 65–75% is deacetylated) and confirmed that chitosanase is more accessible to the chitinous component and more effective for the cell wall digestion than chitinase.

According to the manufacture’s instruction, the reagent ‘calcofluor white’ used for the observation of the mycelial cells by fluorescence microscopy stains chitin and cellulose. It has been often used for the staining of fungal cells including yeast. The whole mycelial cells of the *Fusarium* pathogen strain M2-1 stained with the fluorescent dye appeared light blue and the mycelial septa emitted stronger light-green emission, suggesting the richness of chitin in the septa. Fluorescence microscopy of the *Fusarium* cells co-incubated with strain H110 or TB8 in the PYG medium at 25 °C clearly indicated severe degradation of the mycelial cells at the early period of co-incubation. Both the strains degraded the mycelial cells uniformly irrespective of the parts of mycelia (hypha or septa), and destroyed them thoroughly. Although any of the two strains (H110 or TB8) did not decompose chitin (Ueki et al. 2017), they decomposed the whole mycelia almost completely. The result suggests that chitosanase produced by these strains definitely attacks the deacetylated parts of chitin and decomposes the cell wall rapidly in the coordination with β-1,3-glucanase as the decomposer of the major cell wall component.

Strains H110 and TB8 decomposed the cell wall sample prepared from the cultured biomass of the *Fusarium* pathogen strain M2-1 as a substrate for the anaerobic cultivation (Ueki et al. 2017). In this study, it was shown that the freeze-dried dead whole biomass of the pathogen also supported good growth of the strains H110 and TB8. The result indicates that these strains have abilities to decompose the fungal biomass regardless of live or death state of the fungus. The time course study of the cultivation of the strains with the fungal biomass showed that both β-1,3-glucanase and chitosanase activities rapidly increased at the early period of the cultivation in H110, whereas a lag period of more than a week was necessary for the active degradation of the biomass by TB8. This was shown by the increase in the products and enzyme activities as well as the degradation of cells observed by fluorescence microscopy. Both the strains can utilize glucosamine and GlcNAc as growth substrate as shown previously (Ueki et al. 2017). Thus, both strains should uptake the monomers (such as glucose, glucosamine or GlcNAc), or oligomers of β-1,3-glucan or chitosan released by decomposition of the fungal biomass with the help of extracellular enzymes. Growth or increase in the products by the strain TB8 was in accordance with the increase of β-1,3-glucanase and chitosanase activities, suggesting that low molecular compounds released from the fungal degradation supported the late growth and production of enzymes. Both the activities of strain TB8 with the fungal biomass were lower than that of strain H110, indicating that strain H110 has an advantage to directly attack the fungal biomass. The result also showed that the high enzyme activities once produced were retained until the end of the 3-week incubation period irrespective of the strains and enzymes. This suggests that the enzymes produced by the anaerobic bacteria during the early period by decomposition of organic matter incorporated into the soil may be stable throughout the period of the treatment, though the enzymatic proteins are possible to be degraded by other microbes in the soil.

When strains H110 and TB8 were cultivated with insoluble substrates like curdlan and the fungal biomass, accurate measurement of their growth was difficult through the measurement of culture turbidity. Thus, the amounts of products such as VFAs or alcohols in the culture supernatants were used as the indicators to know the degree of growth of the bacteria in this study. The results obtained by the cultivation with the fungal biomass showed that the amounts of products roughly corresponded with the enzyme activities detected in the cultures. The amounts of fermentation products and the enzyme activities in the culture supernatants with dry yeast as the substrate suggested that both anaerobic strains have the ability to degrade the yeast biomass similarly as the *Fusarium* biomass.

Wheat bran and *Brassica* plants are common materials incorporated into soil for the BSD-treatments. The anaerobic strains examined in this study were actually isolated from the BSD-treated soil samples using either material. Wheat barn, judging from the amounts of products, was found to be a useful material to support their active growth. Under the presence of wheat bran, both strains grew and brought about death of the *Fusarium* pathogen at least by 7th day of the co-incubation. *Brassica* leaves also appeared to have a potential to support growth of these anaerobes. High enzyme activities detected in both strains during the co-incubation with wheat bran coincided well with the degradation of the fungal cells observed by fluorescence microscopy and death of the pathogen. Wheat bran generally contains starch and non-starch polysaccharides like xylan, mainly composed of arabinose and xylose as the major components (Kabel et al. 2002). Strains H110 and TB8 utilize starch and arabinose as growth substrates (Ueki et al. 2017). Thus, wheat bran seems to provide effective substrates for growth of these anaerobic bacteria resulting in active production of the antifungal enzymes. The β-1,3-glucanase activities of strain H110 were usually higher than those of strain TB8 for almost all substrates examined. However, strain TB8 showed higher β-1,3-glucanase activity than that of strain H110 with wheat bran and killed the *Fusarium* pathogen promptly. Although strains H110 and TB8 were both identified as *C. beijerinckii*, the physiological properties or activities under the treatments might be different according to the actual conditions in soil.

The two enzymes detected in this study may synergistically act on the cell wall structure, mainly composed of glucan, chitin, or chitosan, and destruct the fungal cells almost completely under the anaerobic conditions. The properties of the enzymes and their strict functions in degradation of fungal cells as well as the genes encoding these enzymes should be investigated as the future studies. Production of other enzymes by strains H110 and TB8 involved in the inactivation of the pathogenic fungi should also be investigated.

## Data Availability

*Clostridium beijerinckii* strains H110 and TB8 are deposited in NBRC (NITE Biological Resource Center, Kisarazu, Japan) as NBRC 112101 and NBRC 112094, respectively. The DDBJ/GenBank accession numbers of 16S rRNA gene sequences of strains H110 and TB8 are LC020492 and LC020493, respectively.
